# Generation of brilliant green fluorescent petunia plants by using a new and potent fluorescent protein transgene

**DOI:** 10.1038/s41598-018-34837-2

**Published:** 2018-11-08

**Authors:** Dong Poh Chin, Ikuo Shiratori, Akihisa Shimizu, Ko Kato, Masahiro Mii, Iwao Waga

**Affiliations:** 1 Center for Environment, Health and Field Sciences, Chiba University, 6-2-1, Kashiwanoha, Kashiwa, Chiba, 277-0882 Japan; 2 Innovation Laboratories, NEC Solution Innovators, Ltd., 1-18-7, Shinkiba, Koto-ku, Tokyo, 136-8627 Japan; 3 Department of Science and Technology, Nara Institute of Science and Technology, 8916-5, Takayama-cho Ikoma, Nara, 630-0192 Japan

## Abstract

The application of fluorescent proteins in ornamental plants has lagged behind despite the recent development of powerful genetic tools. Although we previously generated transgenic torenia plants expressing green fluorescent protein from marine plankton (CpYGFP), in which bright fluorescence was easily visible at the whole plant level, the maximum excitation of this protein within the visible light spectrum required the use of a coloured emission filter to eliminate exciting light. Here, to overcome this limitation, we generated transgenic petunia plants expressing eYGFPuv, a CpYGFP derivative exhibiting bright fluorescence under invisible ultraviolet (UV) light excitation, with a novel combination of transcriptional terminator plus translational enhancer. As expected, all transgenic plants exhibited brilliant green fluorescence easily visible to the naked eye without an emission filter. In addition, fluorescence expressed in transgenic petunia flowers was stable during long-term vegetative propagation. Finally, we visually and quantitatively confirmed that transgenic petunia flowers resist to long-term exposure of UV without any damages such as fluorescence decay and withering. Thus, our whole-plant fluorescence imaging tool, that does not require high sensitive imaging equipment or special imaging conditions for observation, might be useful not only for basic plant research but also for ornamental purposes as a novel flower property.

## Introduction

Fluorescent proteins (FPs) have become essential tools for studying gene regulation and protein localization, and for live imaging of molecular interactions^1,2^. Furthermore, FPs have a variety of creative applications, ranging from new aquarium fish (https://www.glofish.com/)^3,4^ to wedding dresses incorporating fibers produced by FP-expressing silkworms^5^. On the contrary, ornamental applications of FPs in plants have lagged behind despite the recent development of powerful genetic tools. Indeed, fluorescent proteins have been reported as a valuable tool for various plant research at the cellular level, in genetic studies as indicators of zygosity, and in the ecological monitoring of genetically modified (GM) plants by analysing the possibility of hybridization with relative species and introgression status^6–9^. However, plants exhibit considerable autofluorescence derived from fluorescent compounds like chlorophyll, flavonols, and/or flavone, which overlap the emission wavelength of most FPs^10,11^. Thus, appropriate optical filters are required to avoid detecting undesirable fluorescence. In addition, generation of fluorescence in plants, visible at the tissue or whole plant level, was exclusively limited to some crops and laboratory strains of *Arabidopsis thaliana* and tobacco plants^6–9,12–17^, while the generation of fluoresce in ornamental plants has remained controversial. Although fluorescent *Eustoma grandiflorum* and *Osteospermum ecklonis* have been generated, their detailed characterization such as transgene copy number and fluorescence properties including resistance to illumination by ultraviolet (UV) light has not been performed^18^.

Our colleagues previously identified a green fluorescence protein (GFP)-like protein derived from the marine copepod *Chiridius poppei* (CpYGFP) which exhibited extremely bright fluorescence and has maximum excitation and emission at 508 nm and 518 nm, respectively^19,20^. We, and other colleagues, also succeeded in generating *Torenia fournieri* and *Chrysanthemum morifolium* flowers expressing CpYGFP, in which bright fluorescence was clearly visible at the whole-plant level under blue light excitation^21,22^. However, CpYGFP exhibited an excitation maximum within the visible light spectrum; hence, like most GFP derivatives, CpYGFP requires a coloured emission filter to eliminate exciting light and to enhance optical contrast.

In fact, the need of emission filters is a major drawback, especially in ornamental applications of FPs. Because the emission energy is partially cut off in most cases, a filter accessory is required, and, because most emission filters have colour, the perception of the labelled object is different from that under natural light^8,9,21^. In addition to the emission filter, any leakage of the visible excitation light might interfere with the dark staging conditions required for ornamental display. Therefore, the present study aimed to generate ornamental plants with highly fluorescent flowers in which fluorescence is efficiently excited by solid-coloured (invisible) light, specifically UV light, and is clearly visualized at the whole plant level without an emission filter. These fluorescent flowers should accelerate the application of FPs to ornamental purposes. To develop such flowers, we employed *eYGFPuv*, a new and potent fluorescent protein transgene, which was recently developed as a CpYGFP derivative exhibiting bright fluorescence with maximum excitation under UV wavelength (398 nm) and a long Stokes shift (104 nm)^23^. Furthermore, eYGFPuv uniquely kept stable fluorescence even under acidic conditions (pKa = 3.0), which were unusual compared to other FPs such as enhanced green fluorescent protein (EGFP) (pKa = 5.7)^23^.

In the present study, we designed an expression cassette in which the coding sequence of *eYGFPuv* was driven by the *cauliflower mosaic virus* (CaMV) 35S promoter combined with the transcriptional terminator of the *heat shock protein 18.2* (HSP) gene of *A. thaliana* (HSP-T878)^24^ and the 5′ untranslated region (5′UTR) of *cold-regulated 47* (COR47) in *A. thaliana* (COR47-5′UTR)^25^. HSP-878 and COR 47-5′UTR have been reported to increase the expression level of the transgene compared to other transcriptional terminators and translational enhancer, and high accumulation of FPs is expected. We introduced a tandemly triplicated expression cassette into commercial strains of garden petunia, *Petunia hybrida* by *Agrobacterium tumefaciens*-mediated transformation and generated fluorescent transgenic (*Tg*) lines expressing eYGFPuv. As expected, *Tg* plants exhibited very bright green fluorescence easily visible to the naked eye under UV excitation, and this fluorescence was kept stable by vegetative propagation. Notably, all fluorescence images presented in this report were taken with short exposure time (≤2 s). In addition, we visually and quantitatively confirmed that transformed *P. hybrida* plants exhibited a certain resistance to long exposure to UV-A irradiation (>2 weeks) without any fluorescence decay, growth inhibition, or morphological changes, albeit some deleterious effects of UV radiation on plants have been reported^26,27^. Thus, the results obtained here strongly suggest that our novel genetic tool for whole- plant fluorescence imaging might be well suited to various ornamental applications of fluorescence in plants, also allowing detailed examinations of protein dynamics, function, or interactions in regulatory and developmental processes in a nondestructive manner.

## Results

### Generation of fluorescent petunia flowers

**Figure 1:**
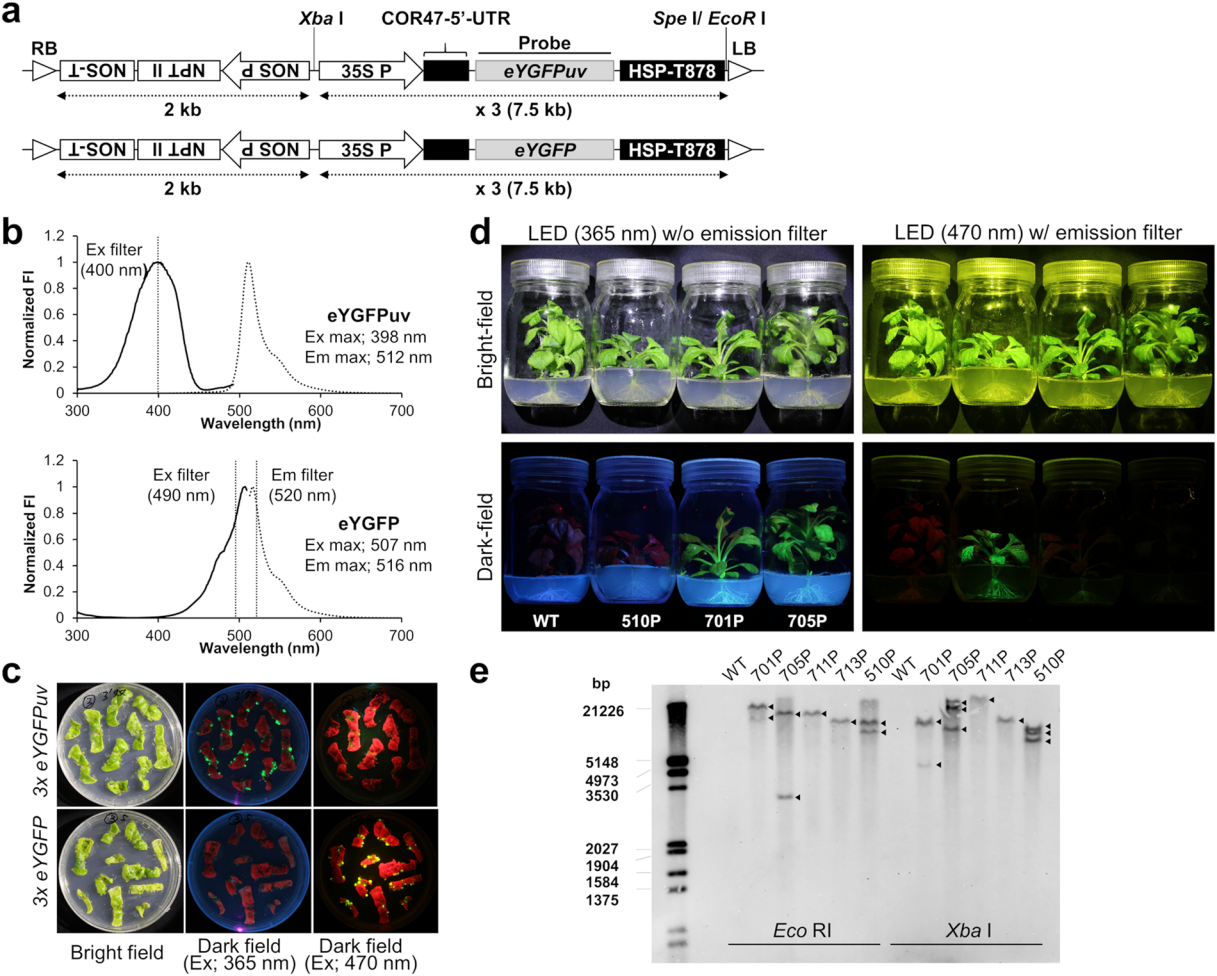
Transformation of petunia plants using *eYGFPuv* or *eYGFP* expression vectors. (**a**) Schematic representation of the expression cassettes cloned into the binary vector pRI909. RB, right border; NOS-T, *nopaline synthase* terminator; NPT II, *neomycin phosphotransferase* gene for kanamycin resistance; NOS P, NOS promoter from *Rhizobium radiobacter*; 35S P, 35S promoter from *cauliflower mosaic virus* (CaMV); COR47, *cold-regulated 47*; HSP-T878, modified *heat shock protein 18.2* gene terminator; LB, left border. (**b**) Fluorescence intensity (FI) excitation (Ex) and emission (Em) spectra of eYGFPuv and eYGFP. Polyhistidine-tagged fluorescent proteins (FPs) were expressed in *Escherichia coli* and purified. Fluorescent proteins were diluted to 2 μM in phosphate buffered saline (PBS; 20 mM, pH 7.4) and scanned in the M1000 Pro microplate reader (TECAN, Männedorf, Switzerland). Normalized Ex (solid line) and Em (dotted line) spectra are shown with Ex and Em maxima, respectively. The threshold value of Ex and Em filters at which light transmittance was approximately 50% are also shown. (**c**) Fluorescence of eYGFPuv and eYGFP observed in calli (**c**) and in juvenile plants grown from acclimated shoots (**d**) photographed using a yellowish emission filter (SC-52, Fujifilm, Tokyo, Japan) for blue light excitation. Fluorescence signals excited by UV or blue LED lights were visually evaluated by taking the photographs by a Canon EOS Kiss Digital X7i cameras as described in Materials and Methods. The image acquisition conditions were summarized in Supplementary Table S2. (**e**) Southern blot analysis of eYFPuv- and eYGFP-expressed *Petunia hybrida* lines generated in this study. Genomic DNA was extracted from the leaves of vegetative-propagated plants and the coding region of *eYGFPuv* was used as the DNA probe. Arrows indicate the positions of *eYGFPuv* and *eYGFP* bands after digestion by *Eco* RI or *Xba* I in lines 701P, 705P, 711P, 713P, and 510P. All experiments mentioned above were performed once.

The present study aimed to generate flowers in which fluorescence is efficiently excited by solid coloured light (i.e., UV light) and clearly observed at the whole plant level without using an emission filter. Therefore, we designed a tandemly triplicated expression cassette using the approach of Sasaki *et al*.^21^ with some modifications (Fig. 1a). The fluorescence excitation and emission spectra of eYGFPuv was compared to that of eYGFP, which was also CpYGFP derivative showing the same fluorescence spectrum as CpYGFP (Fig. 1b)^23^. For reference, the sequences, structure, and detailed fluorescence properties of each fluorescent protein were briefly reproduced in supplementary information (Supplementary Figure S1 and Supplementary Table S1)^23^. We first examined the intracellular localizations of eYGFPuv and eYGFP stably expressed in tobacco BY-2 cells and confirmed that the fluorescence of these FPs, as well as that of intact CpYGFP, was mainly observed in the cytoplasm with no preferential localization to the nucleus or vacuole (Supplementary Figure S2). In addition, microscopic analysis revealed that the fluorescence of BY-2 cells expressing the sextuplicated *eYGFP* trensgene (6 × *eYGFP*) rarely differed from that expressing the 3 × *eYGFP* gene (Supplementary Figure S2).

The strong fluorescence provided by transgenes emerged at an early stage of transformation. When 3 × *eYGFPuv* or 3 × *eYGFP* transgene were introduced into ‘Creepia White’ *P. hybrida* by *A. tumefaciens*-mediated transformation, many of the putative transformed calli generated on the leaf disks exhibited bright fluorescence (Fig. 1c). As expected, under solid-coloured UV excitation (365 nm), only eYGFPuv-expressing calli exhibited substantial fluorescence; eYGFP fluorescence was only clearly visible when excited by blue visible light (470 nm). Transformed juvenile plants regenerated from these calli kept brilliant florescence at the whole plant level, including stem and root fluorescence (Fig. 1d). Under UV excitation, only eYGFPuv fluorescence was clearly visible, while eYGFP fluorescence was clearly visible only when excited by blue light. Notably, eYGFP fluorescence in the 510 P line was extremely bright and observed at the whole plant level, although its expression in leaves was not entirely uniform but somewhat tessellated. Nevertheless, four of the 18 independent transgenic lines expressing eYGFPuv (701P, 705P, 711P, and 713P) and one of the three eYGFP-expressing line (510P) exhibited particularly bright fluorescence and were selected for further analysis.

Next, we examined the copy numbers of these transgenes by southern blot analysis (Fig. 1e). Both *Eco* RI and *Xba* I cut behind the probe sequence and distinguishes the head-tail tandem insertion. Unfortunately, the number of bands was not completely identical between *Eco* RI-cut samples and *Xba* I-cut samples; however, at least, we confirmed that 711P and 713P had only one transgene copy, while 701P, 705P, and 510P had more than two transgene copies.

### Observation of fluorescence in transgenic *P. hybrida* plants

**Figure 2:**
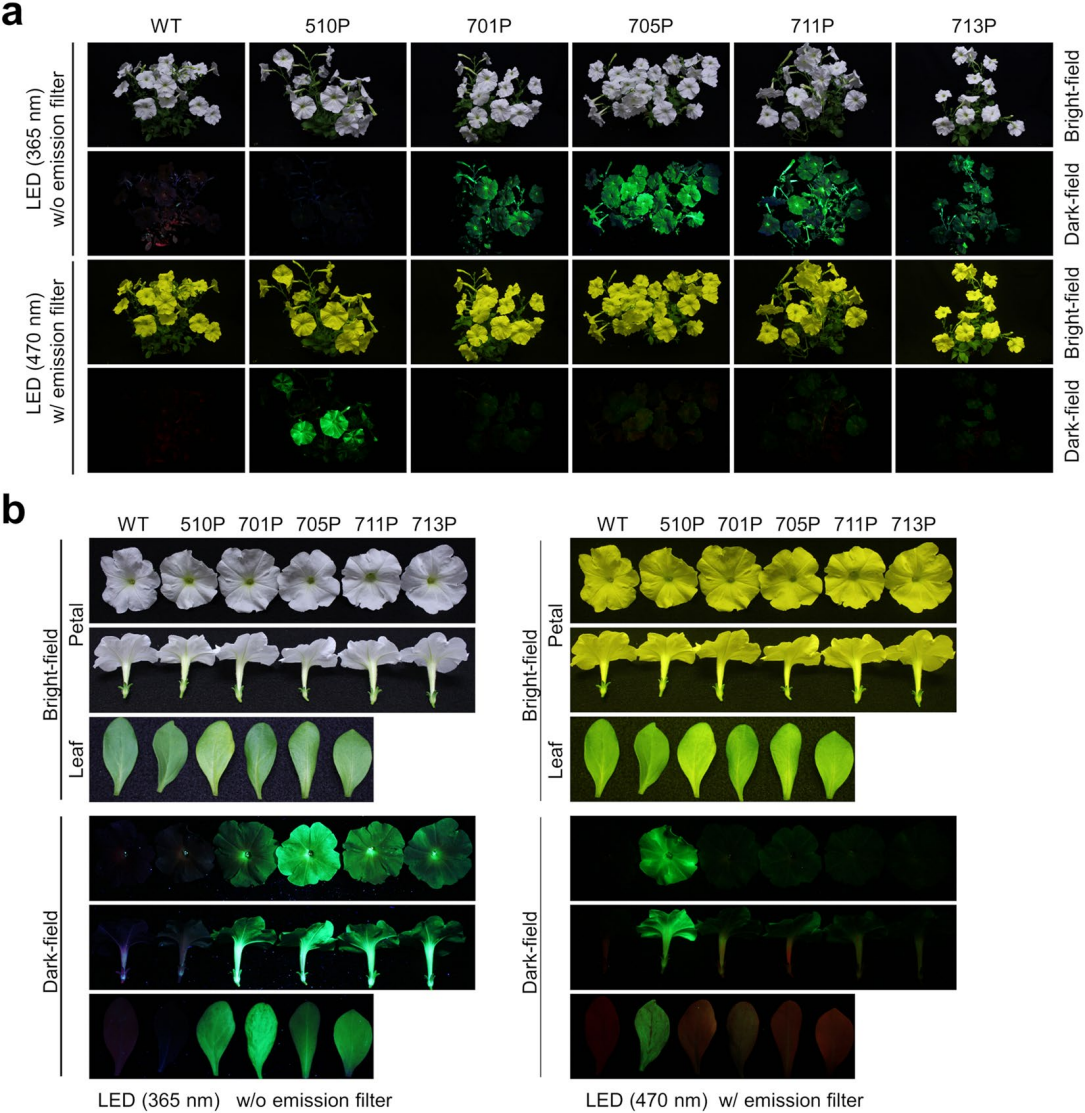
Fluorescence in transgenic *Petunia hybrida* plants from several lineages. (**a**) Whole plant images of wild type (WT) ‘Creepia White’ plants and transgenic (*Tg*) plants expressing eYGFPuv (701P, 705P, 711P, and 713P) or eYGFP (510P) taken under ultraviolet (UV) light excitation (365 nm) or blue light excitation (470 nm). To compare fluorescence intensity among *Tg* flowers, photographs were taken under the same conditions (described in Supplementary Table S2). (**b**) Petals and leaves of WT and *Tg* plants were harvested and placed parallel to each other. Each image was taken under UV or blue light excitation as described above. In all experiments, the fluorescence excited by blue visible light was observed through a yellowish emission filter (SC-52, Fujifilm, Tokyo, Japan). A representative of two independent experiments using different pots is shown.

**Figure 3:**
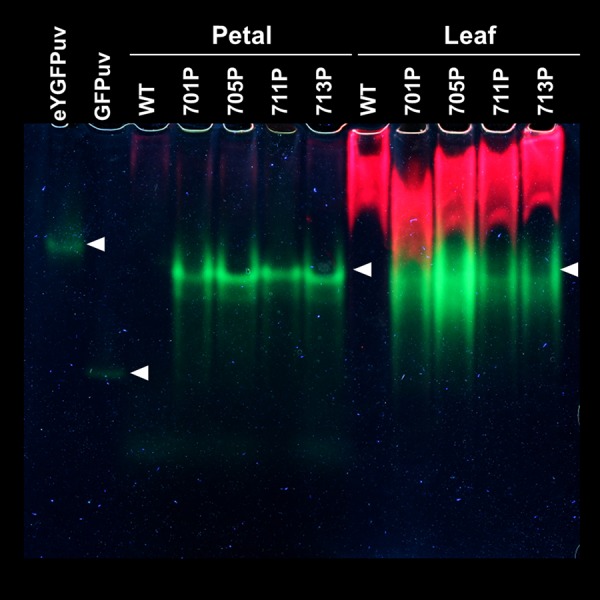
Native polyacrylamide gel electrophoresis of crude protein extracts from transgenic *Petunia hybrida* flowers. Two µg of polyhistidine-tagged eYGFPuv and GFPuv proteins and 80 µg of crude protein extracts from petals and leaves of wild type or eYGFPuv-expressing plants (701P, 705P, 711P, and 713P) were loaded on 10% polyacrylamide gels. Images were obtained by taking photographs under UV LED lights as described in Materials and methods. The image acquisition condition was listed in Supplementary Table S2. While eYGFPuv (theoretical molecular weight (MW): 25.9 kDa) forms dimers, commercial GFPuv (theoretical MW: 28.0 kDa) retains monomeric forms under normal physiological conditions^23^. The experiments were repeated twice with similar results, and one representative result is shown.

Because one of the main purposes of this study was to obtain fluorescent flowers suitable for visual observation-based experiments or for display, we first evaluated fluorescence intensity using photographs, as this is the simplest and most effective method. We compared the fluorescence of adult *P. hybrida* plants expressing eYGFPuv (701P, 705P, 711P, and 713P) with that of WT or eYGFP-expressing 510P plants (Fig. 2a). As expected, when excited by UV light, eYGFPuv-expressing plants, especially 705P, exhibited strong florescence at the whole plant level, including petals, leaves, and stems, and this fluorescence was much stronger than that observed when the same plants were excited by blue visible light. On the other hand, 510P only exhibited bright and specific fluorescence when excited by blue visible light, which was observed using a yellowish emission filter. The fluorescence of harvested petals and leaves is shown in Fig. 2b. Under UV excitation, only eYGFPuv-expressing petals and leaves, especially those derived from 705P, exhibited very bright green fluorescence, while under blue light excitation, only eYGFP-expressing 510P exhibited bright fluorescence. Notably, the fluorescence expressed in 510P petals was much stronger than that expressed in leaves. The fluorescence pattern of eYGFP-expressing 510P petals was almost uniform when compared with that of leaves, and this was relatively less tessellated than that of juvenile plants (Fig. 1d). Crude protein extracts from petals and leaves of WT and eYGFPuv-expressing transgenic lines analysed by native-PAGE revealed strong fluorescence signals of the corresponding protein band, which were in agreement with the patterns observed in visual inspection under UV light excitation (Fig. 3).

**Figure 4:**
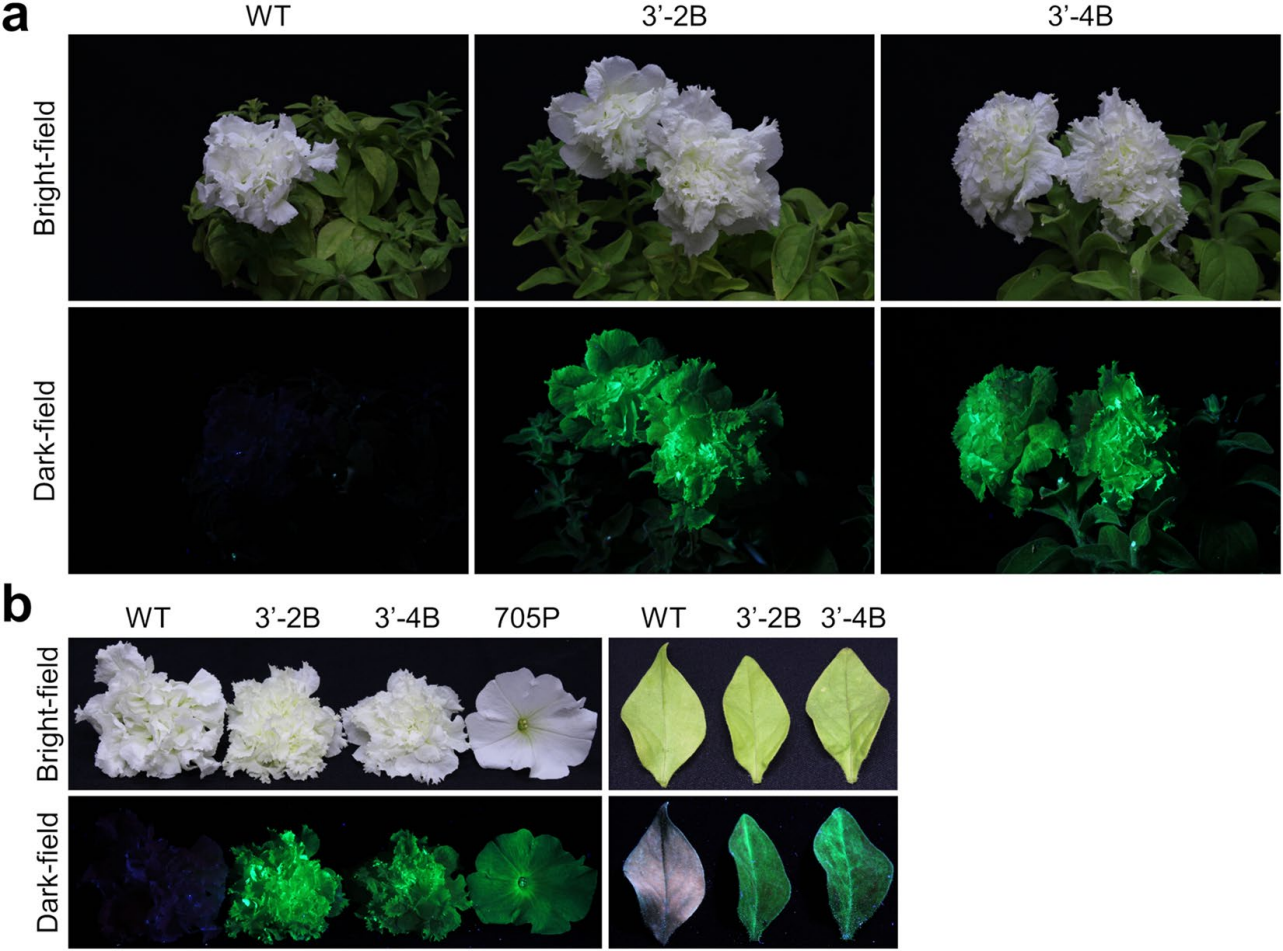
Generation of *Petunia hybrida* lineages displaying double flowers expressing eYGFPuv. (**a**) Whole plant images of wild type (WT) ‘Dressup Neo’ and fluorescent transgenic (*Tg*) plants expressing eYGFPuv (3′-2B and 3′-4B) taken under UV light excitation. To compare fluorescence intensity among *Tg* flowers, photographs were taken under the same conditions (described in Supplementary Table S2). (**b**) Petals and leaves of WT and *Tg* plants were harvested and placed parallel to each other. Each image was taken under UV excitation as described above. A representative of two independent experiments using different pots is shown.

Because of the self-incompatible nature of the F1 hybrid cultivar, we had to maintain *Tg* lines by vegetative propagation. However, the bright fluorescence of *Tg* flowers was kept for at least 18 months, i.e., from the beginning of shoot formation until the preparation of this manuscript. A cultivar of *P. hybrid*, ‘Dressup Neo’ displaying double flowers, which was more glamorous and luxurious than the cultivars displaying single flowers, was also used as host to generate fluorescent flowers expressing eYGFPuv. As expected, under UV excitation, eYGFPuv-expressing plants exhibited very bright green fluorescence with high-contrast at the whole plant level, which was easily visualized with naked eye (Fig. 4a). The fluorescence of harvested petals and leaves exhibited the same tendency, and the fluorescence expressed in 3′-2B and 3′-4B petals were comparable to or brighter than that of the single-flowered lineage 705P (Fig. 4b). Using our novel eYGFPuv gene with a potent combination of transcriptional terminator plus translational enhancer, we could generate green fluorescent flowers in which bright fluorescence can be easily visualized at the whole plant level without any high sensitive imaging equipment and emission filter.

### Levels of mRNA and protein expression in transgenic *P. hybrida* plants

Because all *Tg* plants tested by visual inspection presented intense fluorescence levels, transgene expression in *P. hybrida* lineages was quantified at both transcription and translation levels. According to the recent validation of reference genes in *P. hybrida*^28^, the transcription level of each FP gene was normalized to that of *cyclophilin (CYP)*, as summarized in Supplementary Table S3. Although transcription levels of FP genes were 2- to 4-fold higher in leaves than in petals, the overall transcription levels of FP genes were similar among *Tg P. hybrida* lines (approximately <2-fold). Crude protein extracts obtained from petals and leaves were scanned for their fluorescence spectra over a broad wavelength range (Supplementary Figure S3), and the fluorescence intensity at the peak excitation and emission wavelengths of each FP was then normalized to that of chlorophyll *a* (Supplementary Table S4)^10^. Fluorescence intensities of eYGFPuv-expressing *P. hybrida* lines (701P, 705P, 711P, and 713P) were in agreement with the results obtained by visual inspection (Fig. 2). Notably, fluorescence intensity expressed in the petals of 510 P was approximately 5-fold higher than that of eYGFPuv-expressing lines, while fluorescence intensity expressed in the leaves of 510 P was comparable to that of eYGFPuv-expressing lines.

### Resistance test against long exposure to UV irradiation

**Figure 5:**
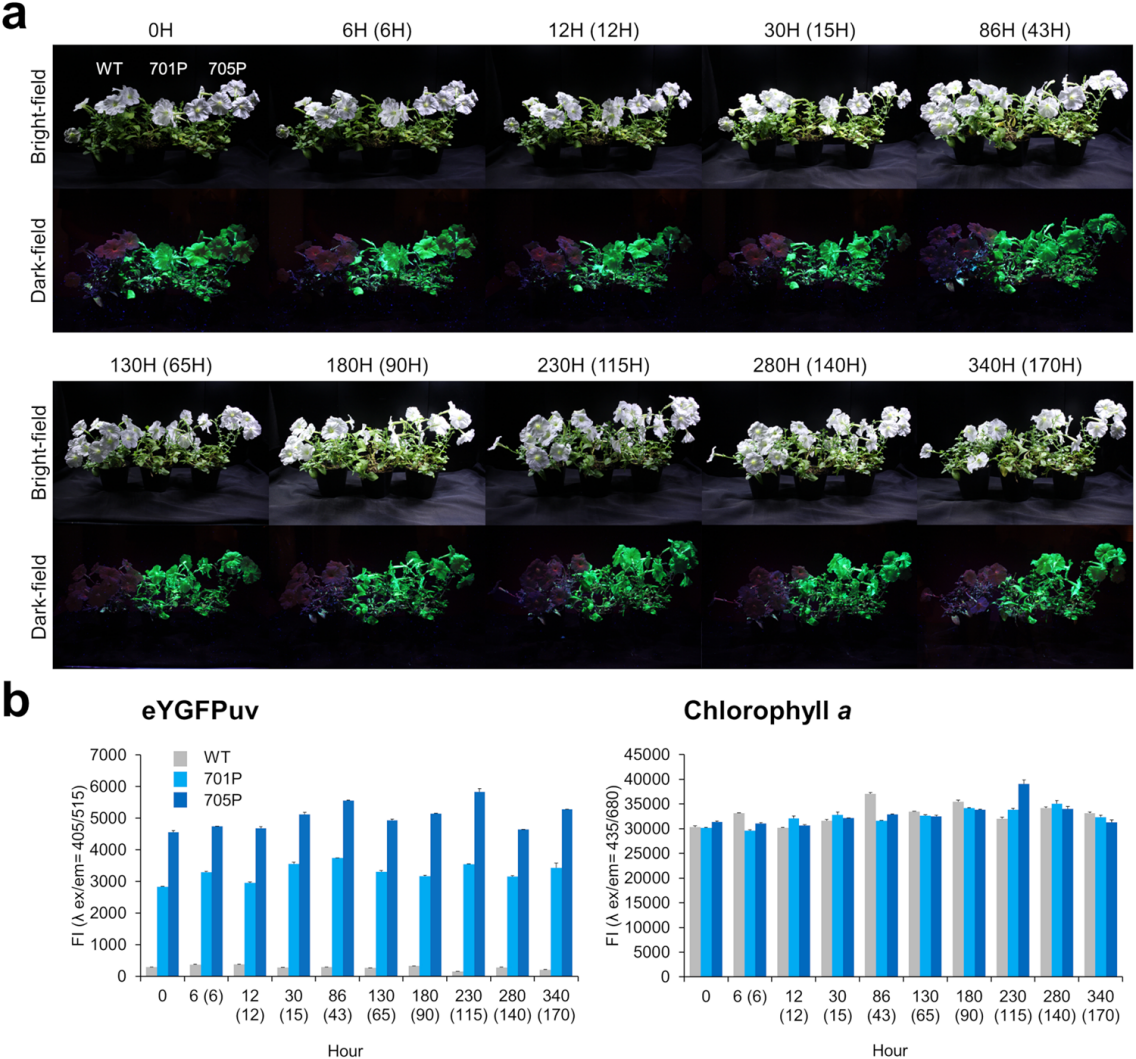
Fluorescent levels of eYGFPuv and chlorophyll *a* during the long-term exposure of *Petunia hybrida* to UV-A light. (**a**) *P. hybrida* flowers (WT, 701P, and 705P) were set in the display case described in Supplementary Figure S4 and exposed to UV irradiation for approximately two weeks (actual UV exposure times are shown in parentheses). At each time point, whole plant images were taken under UV light excitation using the same image acquisition conditions (reported in Supplementary Table S2). The experiments were repeated twice with similar results, and one representative result is shown. (**b**) 4 mg mL^−1^ (50 μL) of crude protein extracts from harvested leaves were scanned in a M1000 Pro reader (TECAN, Männedorf, Switzerland) using the settings described in Supplementary Figure S3 and Supplementary Table S4. Fluorescence intensity (FI) at the peak excitation (ex) and emission (em) wavelengths of eYGFPuv (λ ex/em = 405/515 nm) and chlorophyll *a* (λ ex/em = 435/680 nm) are shown. The experiments were repeated twice with each data point measured in triplicates; a representative data set is shown as the mean value ± standard deviation.

Because UV light is invisible to the human eye, it has certain advantages for the dark staging conditions required for ornamental display, albeit being harmful to most species by causing damage to DNA, RNA, and proteins, and leading to increased production of free radicals that can activate transposons and further mutations^26,27^. In addition, the transfection/transduction of FPs, particularly in animal cells, can cause a photonic disturbance that originates free radicals and eventual oxidative damage^6,8,29^. Regarding plant cells, FPs are now generally accepted not to be cytotoxic^6,8,14^. In addition, no growth inhibition or morphological defect was observed in the fluorescent plants generated in this study (data not shown). However, because resistance to long UV exposure will be important for the application of FPs in ornamental flowers, we examined the expression level of the fluorescence gene and anti-oxidative stress responses in *Tg P. hybrida* flowers during two weeks of exposure to UV-A light. A display case equipped with UV LED lights and white LED grow lights (Supplementary Figure S4) was used to expose WT and eYGFPuv-expressing *P. hybrida* lines (701P and 705P) to UV and white grow lights for 12 h, followed by a 12 h period in total darkness. Notably, all plants, including WT plants, exhibited no specific growth inhibition and withering under UV exposure during the two weeks period (Fig. 5a). Furthermore, we visually (Fig. 5a) and quantitatively (Fig. 5b) confirmed that there was no remarkable fluorescence decay in both eYGFPuv and chlorophyll *a* among harvested leaves, as long as these harvested leaves presented the same green colour and vigour. At the transcriptional level, *eYGFPuv*/*CYP* mRNA ratio was approximately constant (Supplementary Figure S5), although absolute amounts of all gene transcripts tested slightly decreased (<10-fold, data not shown) after 6 h of exposure. We also measured the mRNA levels of genes coding for the typical anti-oxidative enzymes *superoxide dismutase (SOD)* and *catalase (CAT)*^30^. Interestingly, the transcription level of *SOD* in WT was slightly higher than in 701P and 705P plants (<10-fold), and, in contrast to *CAT*, *SOD* expression in all tested plants immediately and transiently increased up to 6 h of UV exposure (Supplementary Figure S4). Overall, these data strongly support that fluorescent *P. hybrida* flowers as well as WT flowers exhibited a certain resistance toward long UV exposure in our experimental settings.

## Discussion

Using our novel fluorescent genes, we have succeeded in generating *P. hybrida* plants exhibiting brilliant green fluorescence, which could be easily visualized at whole plant level without using any high sensitive imaging equipment. Because the excitation maximum of eYGFPuv is within the UV-A region (400 nm)^23^, the fluorescence of these transgenic plants was observed without any emission filter, which constitutes a crucial progress from previously reported fluorescent flowers^21^. Notably, we could obtain bright fluorescent images under unexceptional image acquisition conditions (≤2 s exposure; Supplementary Table S2). Thus, the fluorescence of these transgenic plants was comparable to or brighter than that of other FP-expressing plants reported as visible at the tissue or whole plant level^8,12–18^, although the detailed acquisition conditions of fluorescence images in such reports was not fully available for comparisons, nor did we directly compare the fluorescence of other GFP variants by generating *Tg* plants. Nevertheless, eYGFPuv might be more useful than CpYGFP or eYGFP as it does not require an emission filter, and seems to be an attractive fluorescence reporter gene for plant research, particularly in cases where the spatiotemporal behaviour of genes with very low detectable expression at the organ or whole plant levels need to be monitored.

Interestingly, visual inspections of *P. hybrida* plants revealed that both juvenile and adult plants might exhibit red autofluorescence, possibly derived from chlorophyll *a*, when excited by UV or blue visible light (Figs 1d and 2). However, green fluorescence derived from eYGFPuv or eYGFP remarkably exceeded this reddish fluorescence and thus we rarely observed red or merged yellow fluorescence in eYGFPuv- or eYGFP-expressing tissues when excited by the appropriate light source (Figs 1d and 2). Because the red fluorescence signals of chlorophyll or chlorophyll-protein complexes observed in each leaf were considerably stronger than the corresponding green fluorescence of FPs in both native-PAGE analysis (Fig. 3) and fluorescence scans (Supplementary Figure S3), detailed localization of FPs’ and chlorophyll fluorescence in tissue sections need to be analysed before discussing the preferential absorption of excitation light by these FPs. Visual inspection of the single-flowered *Tg* line 510 P (Creepia White) revealed that the fluorescent pattern of eYGFP in its leaves was somewhat tessellated (Figs 1d and 2) and that the fluorescence expressed in its petals was remarkably stronger than that expressed in its leaves (Fig. 2), suggesting a transgene locus effect. However, it is also possible that the CaMV 35 S promoter did not work constitutively in the *P. hybrida* strains used in this study^7,31,32^.

Bright and stable fluorescence was achieved in the present study in two commercial cultivars of *P. hybrida*, which is a popular bedding plant worldwide (Figs 2 and 4). Because plants generally have weak acid intracellular environment, ranging from approximately pH 4.5 to 7.2^33^, the strong tolerance of eYGFPuv to acidic conditions (pKa = 3.0) might partly attributed to the stability of fluorescence in these plants. In addition, various deleterious effects of UV on the growth, expression of important photosynthetic genes, ribulose-1,5-bisphosphate carboxylase/oxygenase activity, and chlorophyll/carotenoids levels have been reported^27,28^. However, at least in our experimental settings, which were sufficient to observe the bright fluorescence of *Tg* plants with the naked eye, fluorescent *P. hybrida* plants exhibited a certain resistance to long UV exposure without any fluorescence decay and/or specific growth inhibition and withering (Fig. 5). In this regard, because, in our experimental settings, wild type also sufficiently resisted to long UV exposure; thus, comparative experiments by using other plant species and other experimental conditions such as continuous UV exposure without interval need to be analysed before discussing the deleterious effects of UV radiation as well as FP’s protecting effects. Anyway, these data greatly support the application of our fluorescence genes for the development of novel ornamental plants with bright fluorescence. We are currently developing fluorescent flowers in other horticultural plants, e.g., *Phalaenopsis* cultivars, *Matthiola incana*, and double *Cyclamen persicum* using the same expression vector, and, at least at the juvenile stage, all *Tg* plants present a bright fluorescence comparable to that of transgenic *P. hybrida* generated in the present study. Therefore, the expression cassette designed in the present study might provide a new insight into the development of fluorescent plants that are difficult to transform but have high commercial value, although commercializing GM flowers requires the assessment of their biodiversity impact according to domestic laws and The Cartagena Protocol on Biosafety regulations applied within each country.

Finally, in addition to contributing to plant research, fluorescent plants have high potential for cultural and educational purposes by showing an amusing side of science. Indeed, green fluorescent *P. hybrida* flowers have been displayed for three months at a public museum for showing the application of FPs originally derived from a deep-sea copepod crustacean (http://www.kahaku.go.jp/exhibitions/ueno/special/2017/deep-ocean/), and were greatly appreciated by researchers and visitors. Therefore, these fluorescent flowers might also be a good tool to gain public acceptance of GM plants^7,8^.

## Materials and Methods

### Reagents

Unless otherwise mentioned, all enzymes were purchased form Takara Bio Inc. (Shiga, Japan). Polyhistidine-tagged CpYGFP derivatives (eYGFPuv and eYGFP) and GFPuv were prepared as previously described^23^.

### Plant materials

We selected *P. hybrida* as the host plant for the transformation experiments because it is an excellent model species for studying the evolutionary development of floral organs and the biosynthesis of pigments^34,35^. In addition, it seems attractive for commercial use, as it is one of the most popular ornamental and bedding plants with an annual wholesale value exceeding 130 million dollars in the USA alone^36^.

White-flowered *P. hybrida* varieties were selected for generating fluorescent flowers because Sasaki *et al*. reported that some petal pigments significantly act to reduce CpYGFP fluorescence, probably shading the excitation/emission light^21^. Seeds of *P. hybrida* ‘Creepia White’ were purchased from Sakata Seed Co. (http://www.sakata.com) and plants of *P. hybrida* ‘Dressup Neo’ were purchased from Takii Seed (http://www.takii.co.jp). All the plant materials used as hosts in transformation experiments were maintained *in vitro* at 25 °C under a 14 h photoperiod with a light intensity of 50 μmol m^−2^ s^−1^, provided by cool white fluorescent lights (32 W, FHF32EX-N-H, Toshiba lighting & technology corporation, Yokosuka, Japan). Tobacco cultured cells (*Nicotiana tabacum* ‘BY-2’) were cultured in modified Murashige–Skoog medium and genetically transformed using *A. tumefaciens* EHA105, as described previously^37^.

### Plasmid construction

The structure of the expression cassette cloned into the plant binary vector pRI909 (Takara) was similar to 3 × CpYGFP^21^, but its 5′UTR was replaced with COR47′s modified 5′UTR (5′-CAAACATTACTCATTCACAAAACCATCTTAAAGCAACTACACAAGTCTTGAAATTTTCTCATATTTTCTATTTACTATATAAACTTTTAATCAAATCAAGATTAAAG-3′) by the 5′UTR of *alcohol dehydrogenase* in *A. thaliana* (AtADH-5′UTR). In brief, we recently reported that COR47-5′UTR enhanced transgene expression by approximately 2-fold compared with the AtADH-5′UTR in stable transgenic (*Tg*) plants^25^. Here, the sequence on the 3′ extremity of COR47-5′UTR was changed from CU to AG to further improve translation efficiency^38^. The coding region of *CpYGFP* [GenBank (https://www.ncbi.nlm.nih.gov/genbank/) Accession No. AB185173] was replaced with *eYGFPuv* (No. LC217533) or *eYGFP* (No. LC217529)^23^ and the performance of both CpYGFP derivatives (i.e., eYGFPuv and eYGFP) was compared. For reference, eYGFP shows the same fluorescence spectrum as CpYGFP, but it exhibits higher fluorescence intensity compared to CpYGFP when expressed in *Escherichia coli* or human tumour cell lines^23^. After generating the constructs described above (pRI909-COR47-5′UTR:YGFP:HSP-T878), the *Xba* I–*Eco*R I fragment was excised from pRI909-COR47-5′UTR:YGFP:HSP-T878 and inserted into the *Spe* I–*Eco*R I gap of pRI909-COR47-5′UTR:YGFP:HSP-T878 to generate *2* × *YGFP*. *3* × *YGFP* was generated using the same procedure.

### Plant transformation and cultivation

The binary vectors were introduced into *A. tumefaciens* strain EHA105 by electroporation. After transformation of *P. hybrida* leaf discs by this bacterial strain as previously reported^39^, transformed tissues and shoots were visually selected using the green fluorescence of FPs as an indicator, in combination with antibiotic selection, to exclude non-fluorescent escape responses. After regeneration of plants from *Tg* shoots, they were transferred to soil and grown in a glasshouse.

### Southern blot analysis

Genomic DNA was extracted from the leaves of each petunia cultivar using the DNeasy plant Maxi kit (QIAGEN, Venlo, Netherlands), following the manufacturer’s instructions. *Eco* RI and *Xba* I were used to digest 10 μg of *P. hybrida* genomic DNA, which was then separated on a 0.9% agarose gel and transferred to a positively charged nylon membrane (Roche Applied Science, Basel, Switzerland). We used the coding region of *eYGFPuv* as the DNA probe in this analysis, and it was labelled using a PCR DIG probe synthesis kit (Roche Applied Science) and the primers 5′-ATGACAACCTTCAAAATCGAGTCC and 5′-CTACATGTCTCTTGGGGCGCTGTT. Hybridization signals were detected by chemiluminescence using CDP-Star (Roche Applied Science) as the substrate, and visualized with an imaging system (Molecular Imager VersaDoc™ MP 5000, Bio-Rad, Hercules, CA, USA).

### Visual fluorescence comparison

Fluorescence was visually evaluated by comparing the photographs taken by a Canon EOS Kiss Digital X7i camera (Canon, Tokyo, Japan) under UV light excitation (30 W LED, light intensity of 150 μmol m^−2^ s^−1^, peak wavelength 365 nm; NS365-FLB-30WR, Nitride semiconductors Co., Ltd, Tokushima, Japan) or blue light excitation (60 W LED, light intensity of 300 μmol m^−2^ s^−1^, peak wavelength 470 nm; VBL-SL150BB, Valore, Kyoto, Japan). Two excitation filters were used: the UV LED light was covered with a UL360 filter (OMG Co. Ltd, Osaka, Japan) to eliminate visible light, and the blue LED light was covered with a SV0490 filter (Asahi Spectra, Tokyo, Japan) to eliminate long-wavelength light. A yellowish sharp cut filter (SC-52, Fujifilm, Tokyo, Japan) was used as an emission filter for blue light excitation. Both LED light sources were placed approximately 300 mm apart from the uppermost petals of each plant to obtain images under the same conditions (f-ratio, ISO speed, shutter speed), as listed in Supplementary Table S2.

### Fluorescence spectroscopy and native-polyacrylamide gel electrophoresis (PAGE) analysis

For protein extraction from the leaves and petals of each cultivar, we used the 8-min Plant Tissue Total Protein Extraction Kit (101Bio, Palo Alto, CA, USA) with native lysis buffer, following the manufacturer’s instructions. Protein concentration of each extract was determined using a BCA protein assay kit (Pierce Biotechnology, Rockland, IL, USA). Fluorescence excitation and emission of 10 mg mL^−1^ (50 μL) of each protein extract were scanned with a M1000 Pro microplate reader (TECAN, Männedorf, Switzerland). For native-PAGE analysis, 2 µg of purified polyhistidine-tagged proteins and 80 µg of each crude protein extract were loaded on 10% polyacrylamide gels (c-PAGEL C10L; ATTO, Tokyo, Japan) and photograph was taken by using a Canon EOS Kiss Digital X7i camera under UV LED lights as described above. The image acquisition condition was listed in Supplementary Table S2.

### Real-time PCR analysis

Total RNA was immediately prepared from leaves and petals of wild type (WT) plants, and eYGFPuv- and eYGFP-expressing plants using the RNeasy Plant Mini Kit (Qiagen), following the manufacturer’s instructions. First-strand complementary DNAs (cDNAs) were synthesized by the ReverTra Ace qPCR RT Master Mix with gDNA Remover (Toyobo, Osaka, Japan), in accordance with the manufacturer’s instructions. For real-time PCR, we used KOD Dash DNA polymerase (Toyobo) supplemented with SYBYR Gold (Thermo Fisher Scientific, Waltham, MA, USA). Real-time PCR was performed on a CFX96 Real-Time system instrument (Bio-Rad), using the primer pairs listed in Supplementary Table S5. Because most reagents for measuring the activity of anti-oxidative enzymes exhibit a certain fluorescence overlapping that of transgenes^40,41^, we only measured the mRNA levels of genes coding for the typical anti-oxidative enzymes *superoxide dismutase (SOD)* and *catalase (CAT)*^30^. Transcript levels of fluorescence genes and anti-oxidative enzyme genes were normalized according to that of *cyclophilin* (*CYP*), as this gene is more suitable than the conventional *GAPDH* due to its stable expression regardless tissue or cell specificity, morphogenetic process, and environmental conditions^28^.

### Resistance against long UV exposure

We prepared a display case equipped with a timer, two UV LED lights (NS365-FLB-30WR), and two White LED grow lights (24 W, 800 μmol m^−2^ s^−1^, ISLIM-150 × 150-WW, CCS Inc, Kyoto, Japan). Wild type and *P. hybrida* lines expressing eYGFPuv (701P and 705P) were set in the display case and exposed to UV and white LED light for 12 h, followed by 12 h of dark condition. Plants were watered once every two days (100 mL) and, at each measuring time point, two leaves were appropriately harvested to prepare crude protein extracts and total RNA as described above. Whole-plant images were photographed under UV light excitation, and the fluorescence excitation and emission of 4 mg mL^−1^ (50 μL) of each protein extract was scanned with the M1000 Pro microplate reader. The transcription levels of the fluorescence (*eYGFPuv*), anti-oxidative enzymes (*CAT* and *SOD*), and house-keeping (*CYP*) genes were determined by real-time PCR, as described in the previous sections.

## Electronic supplementary material

Supplementary information
