# Supplementary material for: Generation of brilliant green fluorescent petunia plants by using a new and potent fluorescent protein transgene
Source: Sci Rep. 2018 Nov 8;8:16556. doi: 10.1038/s41598-018-34837-2 (PMC6224394; doi:10.1038/s41598-018-34837-2)
Supplement: Supplementary file 1 — Supplementary information [file 41598_2018_34837_MOESM1_ESM.pdf]

Supplementary information for:

Generation of brilliant green fluorescent petunia plants by using a new and potent fluorescent protein transgene

Dong Poh Chin[1, †], Ikuo Shiratori[2, †, *], Akihisa Shimizu[2], Ko Kato[3], Masahiro Mii[1] and Iwao Waga[2]

[1]Center for Environment, Health and Field Sciences, Chiba University, 6-2-1, Kashiwanoha, Kashiwa, Chiba 277-0882, Japan; [2]Innovation Laboratories, NEC Solution Innovators, Ltd., 1-18-7, Shinkiba, Koto-ku, Tokyo 136-8627, Japan; [3]Department of Science and Technology, Nara Institute of Science and Technology, 8916-5, Takayama-cho Ikoma, Nara 630-0192, Japan.

* Correspondence: Ikuo Shiratori, E-mail: iku-shiratori@xc.jp.nec.com; Tel: +81 355342619; Fax: +81 355342620

† These authors contributed equally to this work.

E-mail addresses:

Dong Poh Chin, dpchin@chiba-u.jp; Akihisa Shimizu, a-shimizu@wm.jp.nec.com; Ko Kato, kou@bs.naist.jp; Masahiro Mii, miim@faculty.chiba-u.jp; Iwao Waga, iwa-waga@wm.jp.nec.com

a

b

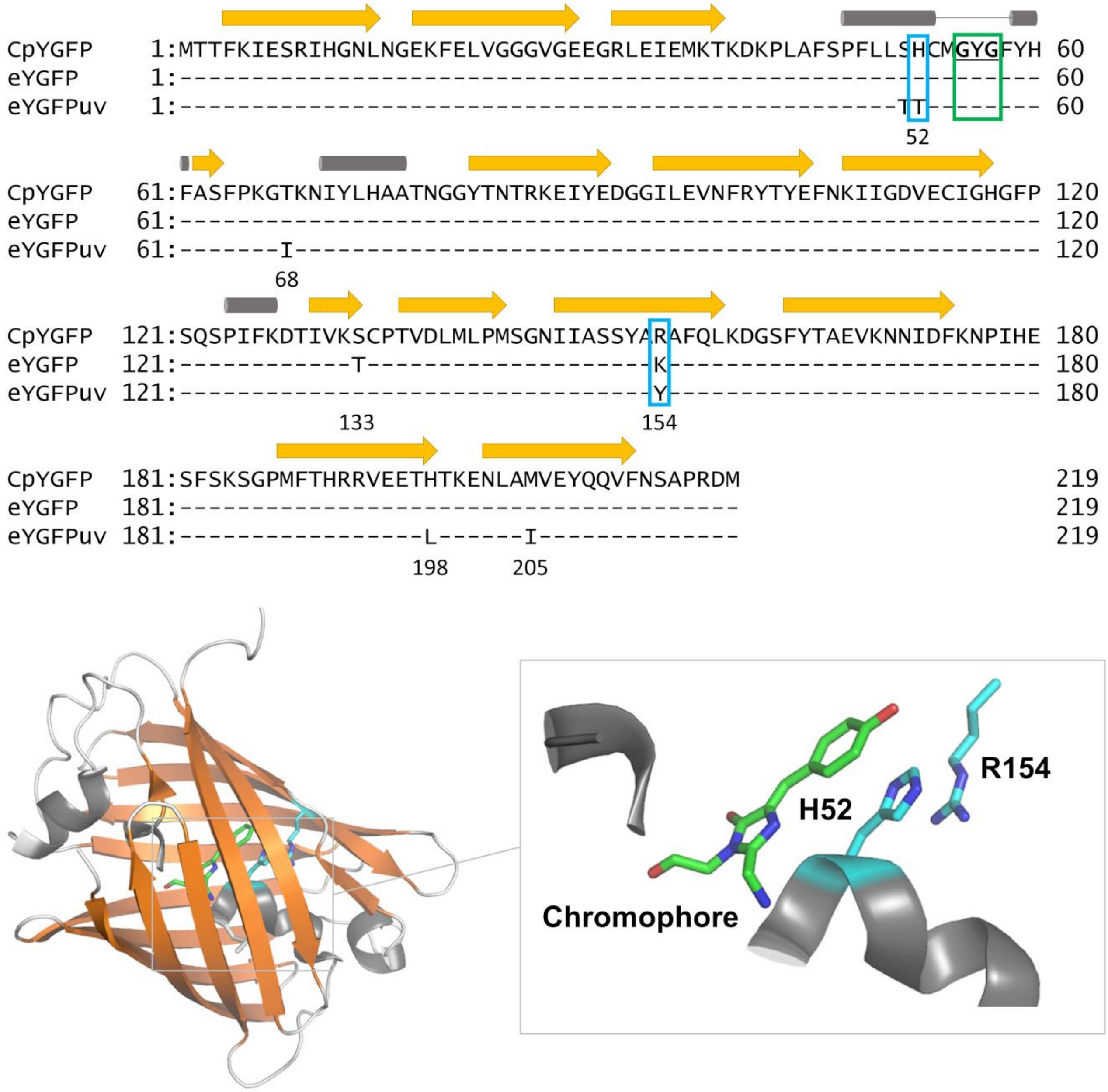

Supplementary Figure S1. Amino acid sequence alignment of CpYGFP, eYGFP and eYGFPuv. (**a**) Secondary structure of CpYGFP and its derivatives are indicated with alpha-helices (gray cylinders) and beta-strands (orange arrows).[20] In comparison with the CpYGFP sequence, there are two and six amino acid replacements in the eYGFP and eYGFPuv, respectively. (**b**) Overall structure of CpYGFP (PDB ID 2DD7). The structure model was drawn using PYMOL software (DeLano Scientific; http://www.pymol.org). Amino acids at positon 52 and R154 are shown with cyan stick. The chromophore (Gly-Tyr-Gly) is colored green. We previously reported that amino acid replacements at position His52 and R154 involved in stacking interaction with the chromophore were likely to alter the hydrogen bond network around the chromophore, and consequently altered their fluorescent characteristics.[23]

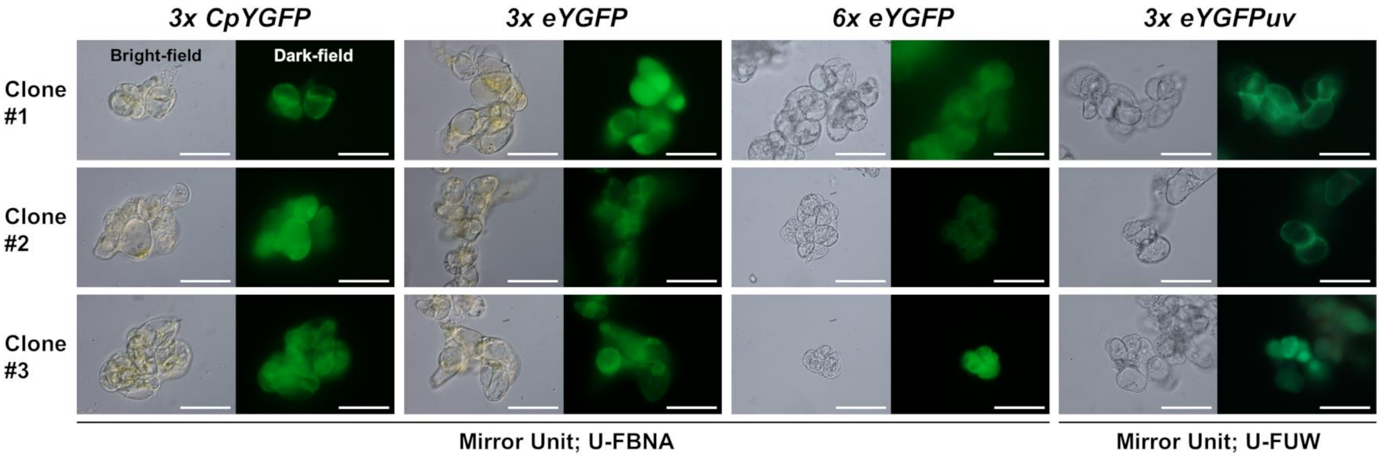

Supplementary Figure S2. Intracellular localization of CpYGFP and its derivatives stably expressed in tobacco BY-2 cells. Suspension culture cells derived from independent calli were observed under a fluorescence BX3 microscope (Olympus, Tokyo, Japan) equipped with a 40× objective and a pair of filters: one for observing blue excitation fluorescence (Olympus Mirror Unit U-FBNA; Excitation filter: 470–495 nm, Emission filter: 510–550 nm, exposure: 100 ms) and another for observing UV excitation fluorescence (Olympus Mirror Unit U-FUW; Excitation filter: 340–390 nm, Emission filter: 420IF, exposure: 200 ms). The experiment was performed once. Scale bar = 50 µm.

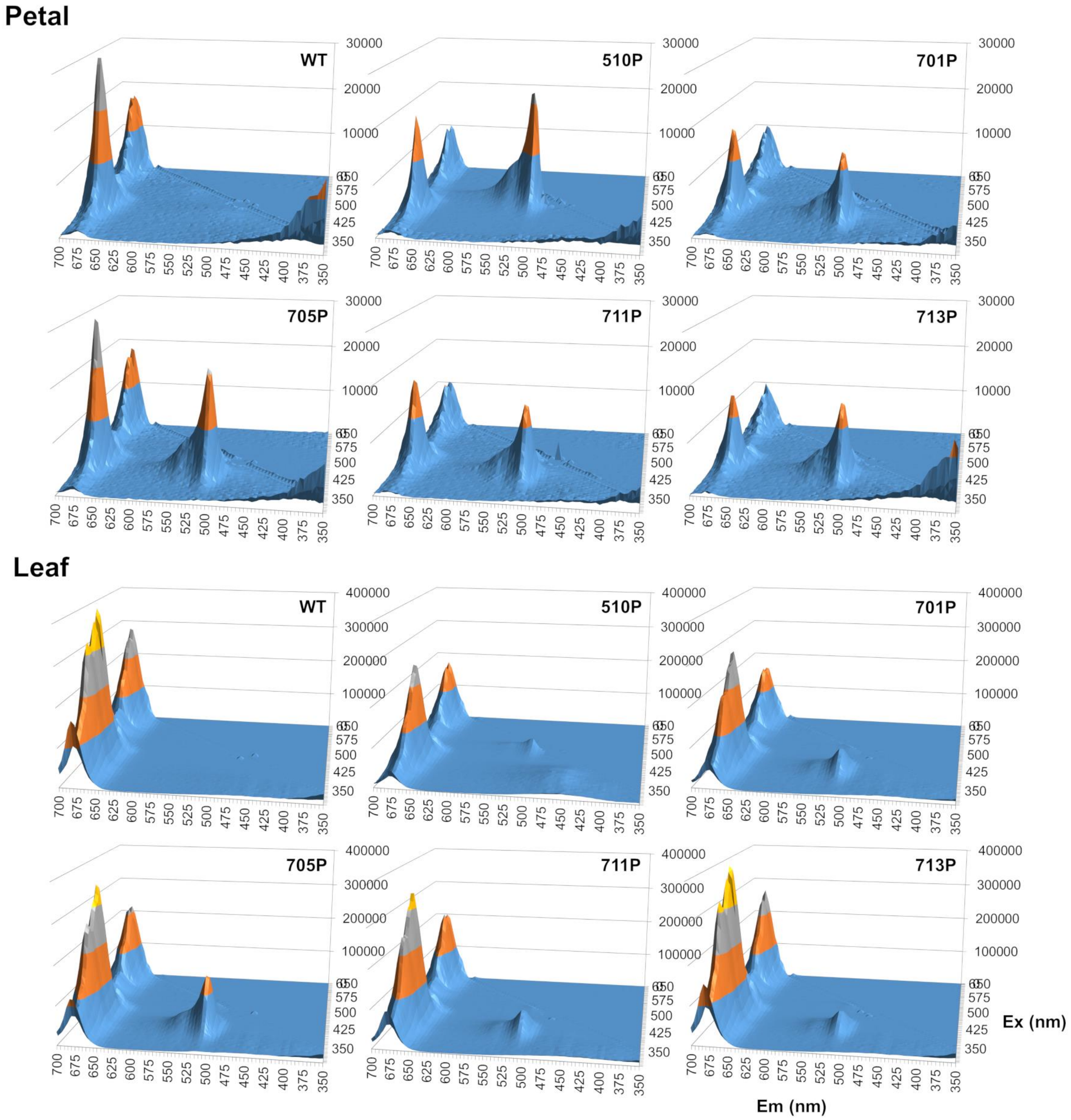

Supplementary Figure S3. Fluorescence scans of crude protein extracts from petals and leaves of wild type and fluorescent petunia plants. 0.5 mg (50 μL) of crude protein extracts from each petal and leaf were scanned in the TECAN M1000 pro microplate reader with the following set of parameters: gain, fixed; step, 5 nm; bandwidth, 5 nm. Notably, the excitation and emission maxima of chlorophyll *a* were 435 nm and 680 nm, respectively. Experiments were repeated twice with similar results, and one representative result is shown.

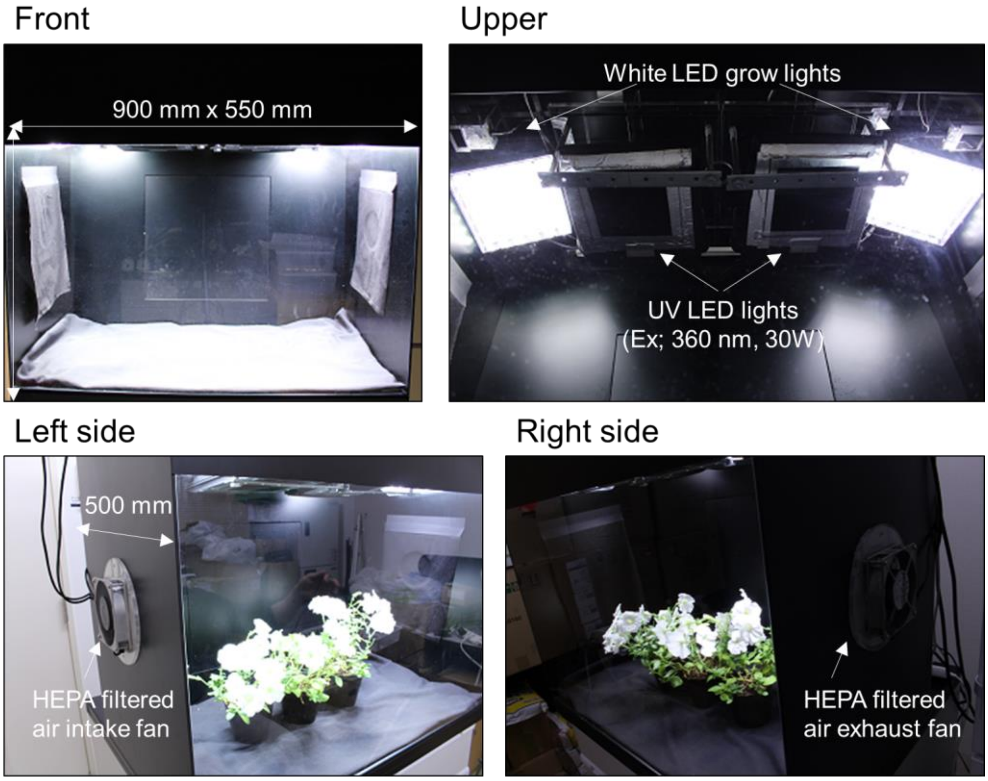

Supplementary Figure S4. Display case used in the resistance test against long UV exposure. HEPA, High efficiency particulate air

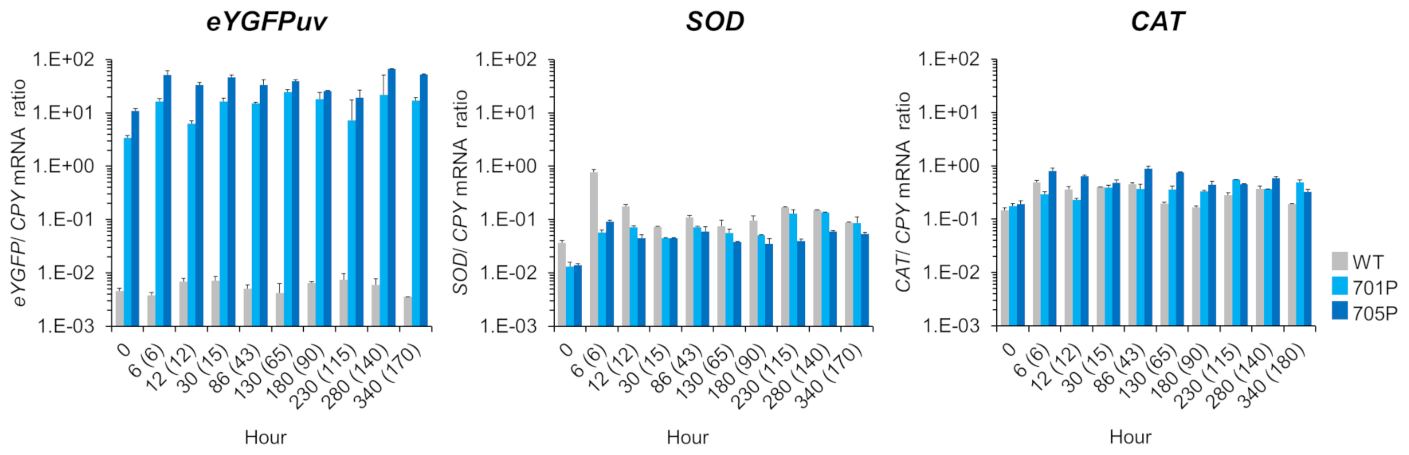

Supplementary Figure S5. Transcription levels of *eYGFPuv*, *superoxide dismutase (SOD)* and *catalase (CAT)* during the UV-resistance test performed for *Petunia hybrida* lineages. *P. hybrida* flowers (wild type, 701P, and 705P) were exposed to UV-A irradiation for approximately two weeks as described in Figure 4 (actual UV exposure times are shown in parentheses). At each time point, we harvested two leaves from each plant and immediately extracted their total RNA. Transcription levels of *eYGFPuv*, *SOD*, and *CAT* were normalized to those of *cyclophilin (CYP)* and these ratios were shown. Experiments were repeated twice with each data point measured in triplicates; a representative data set is shown as the mean value ± standard deviation.

Supplementary Table S1. Fluorescence properties of CpYGFP, eYGFP and eYGFPuv

Gene name	Accession	Ex (nm)	Em (nm)	EC ($M^{-1}cm^{-1}$)	QY	MW (kDa)	Oligomeric states	pKa	Ref
CpYGFP	AB185173	508	518	101,000	0.53	25.9	Dimer	N.D.	[19], [23]
eYGFP	LC217529	507	516	124,000	0.58	25.9	Dimer	3.81	[23]
eYGFPuv	LC217533	398	512	31,200	0.76	25.9	Dimer	3.00	[23]

The excitation (Ex) and emission (Em) wavelengths, molar extinction coefficient (EC), quantum yield (QY), theoretical molecular weight (MW), oligomeric states, pKa, and reference (Ref) are listed.[23] Accessions are shown as identifiers of GenBank (https://www.ncbi.nlm.nih.gov/genbank/). N.D.: not determined.

Supplementary Table S2. Acquisition conditions of fluorescent images (dark-field)

Figure	Description	F ratio	ISO speed	Exposure (sec)	Focal length (mm)
Figure 1c	Upper middle	3.5	1600	1/10	18
	Upper left	3.5	1600	1/10	18
	Lower middle	4	1600	1/10	25
	Lower left	4	1600	1/10	26
Figure 1d	Lower left	11	200	2	43
	Lower right	11	200	2	43
Figure 2a	LED 365 nm	14	800	2	18
	LED 470 nm	16	800	2	21
Figure 2b	LED 365 nm petal (upper)	14	800	2	18
	LED 365 nm petal (lower)	14	800	2	18
	LED 365 nm/ leaf	22	800	2	46
	LED 470 nm/ petal (upper)	14	800	2	18
	LED 470 nm/ petal (lower)	14	800	2	18
	LED 470 nm/ leaf	14	800	2	46
Figure 3	LED 365 nm	5.6	200	1	52
Figure 4a	LED 365 nm	11	400	1	55
Figure 4b	LED 365 nm /Petal	11	400	1	46
	LED 365 nm /Leaf	11	400	2	55
Figure 5a	LED 365 nm	5.6	400	2	21

[1]ISO, International Organization for Standardization

Supplementary Table S3. Transcript levels of the fluorescence gene in the leaves and petals of transgenic *Petunia hybrida* plants in relation to wild type (WT) plants.

	Petal	Leaf
WT	1.0	1.0
510P	1115.7 ±18.0	1906.1 ±6.4
701P	1062.1 ±19.5	3725.9 ±40.7
705P	1844.6 ±31.2	4405.3 ±88.2
511P	1133.0 ±20.4	3995.0 ±60.8
513P	1037.3 ±47.8	3735.9 ±35.4

[1] Primers used for real-time PCR amplification are shown in Supplementary Table S5.

The experiments were repeated twice with each data measured in triplicates; a representative data set is shown as the mean value ± standard deviation.

Supplementary Table S4. Translation levels of the fluorescence gene in leaves and petals of transgenic *Petunia hybrida* plants in relation to wild type (WT) plants.

	Petal		Leaf	
	eYGFPuv (λ ex/em= 405/515)	eYGFP (λ ex/em= 505/520)	eYGFPuv (λ ex/em= 405/515)	eYGFP (λ ex/em= 505/520)
WT	1	1	1	1
510P	29.4 ±2.6	1021.9 ±6.6	6.7 ±0.4	140.2 ±2.4
701P	208.0 ±8.4	97.9 ±5.1	102.8 ±2.6	8.4 ±0.1
705P	196.8 ±0.2	81.7 ±6.3	194.6 ±0.8	9.0 ±4.5
711P	219.8 ±5.5	108.8 ±6.4	81.8 ±3.4	6.8 ±0.2
713P	272.9 ±3.3	118.3 ±0.0	57.6 ±0.2	5.8 ±0.5

[1] 0.5 mg (50 µL) of crude protein extracts from each petal and leave were scanned in a M1000 pro reader (TECAN, Männedorf, Switzerland) under the following parameters: gain, fixed; step, 5 nm; bandwidth: 5 nm. Fluorescence intensity at the peak excitation (ex) and emission (em) wavelengths (in nm) of each fluorescence protein (FP) was normalized to that of chlorophyll *a* (λ ex/em = 435/680 nm). Experiments were repeated twice and data were measured in triplicate; a representative value is shown as the mean ±standard deviation.

Supplementary Table S5. Primer sets used for real-time PCR.

Gene name	Accession	Primer	Sequence (5'-3')	Reference
Cyclophilin (CYP)	SGN-U207595[1]	Forward	AGGCTCATCATTCCACCGTGT	[28]
		Reverse	TCATCTGCGAACTTAGCACCG	
Superoxide dismutase (SOD)	Q43779[2]	Forward	GACCCCATTTCAATCCTAACGG	[30]
		Reverse	AGTGGTAAGGCTGAGTTCGTGGC	
Catalase (CAT)	Q6T2D5[2]	Forward	GCCATGCTACTCAGGACCTC	This work
		Reverse	GCTGCAAAGGCAAAATATCC	
eYGFPuv	LC217533[2]	Forward	TCGTGAAGAGTTGTCCCACG	[23]
		Reverse	TGAGAGTCTCCTCGACACGT	

[1,2]Accessions are shown as identifiers of Solanaceae Genomics Network (https://solgenomics.net/)[1] or GenBank (https://www.ncbi.nlm.nih.gov/genbank/)[2].
